# Low level of attention to health inequalities in prevention planning activities of the Italian Regions

**DOI:** 10.1186/s12939-016-0318-8

**Published:** 2016-02-19

**Authors:** Corrado De Vito, Azzurra Massimi, Domitilla Di Thiene, Annalisa Rosso, Elvira D’Andrea, Maria Rosaria Vacchio, Paolo Villari, Carolina Marzuillo

**Affiliations:** Department of Public Health and Infectious Diseases, Sapienza University of Rome, Piazzale Aldo Moro 5, Rome, 00185 Italy

**Keywords:** Prevention, Health inequalities, National Prevention Plan

## Abstract

**Background:**

Health promotion and prevention activities should tackle health inequalities to reduce disparities in health among disadvantaged populations. This study aimed to assess the extent to which the Italian Regions considered health inequalities during the planning of prevention activities, to detect geographical differences and to identify the possible determinants of differences in attention to health inequalities.

**Methods:**

The 19 Regional Prevention Plans (RPPs) developed by Italian Regions within the National Prevention Plan (NPP) 2010–2013 were assessed using a specific tool to address the level of attention to health inequalities. Univariate and multivariate analyses were performed to identify regional characteristics associated with a higher level of attention to health inequalities.

**Results:**

Of the 702 projects included in the 19 RPPs, only 56 (8.0 %) specifically addressed issues related to health inequalities. The results of the multivariate analysis showed that a higher level of attention was associated with the macroarea of intervention ‘prevention in high-risk groups’, with the higher quality of the Strategic Plan Section of the RPP and with the higher percentage of migrants in the Region in 2010. Moreover, projects that addressed the topic of health inequalities were more likely to be developed in the Northern Regions, in Regions with a lower level of ‘linking social capital’ and with a Higher Regional Health Care Expenditure (RHCE) as a percentage of Regional Gross Domestic Product (RGDP) in 2010.

**Conclusions:**

The level of attention to health inequalities in the regional planning process of prevention activities 2010–2013 in Italy is low. The results of this study supported the new round of prevention planning in Italy, and highlight the urgent need to increase the number of policies and interventions able to reduce health inequalities.

## Background

A comparison of 22 European countries showed that health inequalities associated with socioeconomic status are present everywhere throughout Europe, highlighting the urgent need for public health research to find effective policies and interventions able to reduce health inequalities [1, 2]. The first report on inequalities in health in Italy, published in 1994, found a strong association between illness and conditions of social and economic disadvantage for all health indicators [3]. A study published ten years later reported that mortality in Italy increased with social disadvantage for a wide range of indicators at both the individual (education, social class, income) and geographical (deprivation indexes) levels [4]. In Italy today, the most important behavioral risk factors for chronic diseases are more common in the Southern Regions and among the economically disadvantaged and less educated groups, whose death risk is about 80 % higher than the general population [5].

Addressing health inequalities has become a priority for several high-income countries, with emphasis on health promotion and prevention activities [6–8]. However, the development of interventions specifically aimed at tackling health inequalities is very challenging, given that the evidence prompting their development is mainly descriptive and that the interventions themselves are focused on modifying lifestyle factors [9, 10]. A recent umbrella review that synthesized the results of thirty systematic reviews of the effects on health inequalities of any intervention based on wider social determinants of health (e.g., housing, living environment, working conditions, etc.) found unclear effects of these interventions, and called for more studies on this topic [9]. Nevertheless, positive effects on the health of disadvantaged individuals have resulted from interventions aimed at reducing exposure to risk factors in the whole population [11–13].

The National Prevention Plan (NPP) is the main policy and planning instrument for prevention in Italy. Issued approximately every 3–5 years, the NPP is the part of the National Health Plan (NHP), which is committed to the development of health promotion and disease prevention activities [14]. In accordance with the Italian institutional framework of healthcare decentralization, the NPP 2010–2012 (extended to 2013) determined that each Italian Region should develop its own Regional Prevention Plan (RPP), designing projects coherent with the regional epidemiological and organizational context. RPPs and their projects offer a unique opportunity to assess the extent to which the Italian Regions considered health inequalities during the planning of prevention activities. The specific objectives of this study were to assess the level of attention to inequalities in RPP projects, to detect geographical differences and to identify the possible determinants of differences in attention to health inequalities.

## Methods

This study is part of a wider project funded by the Italian Ministry of Health aimed at identifying strengths and weaknesses of the prevention planning process in Italy, and at providing suggestions for strengthening regional capacities. These outcomes should prove useful for subsequent rounds of prevention planning.

A tool specifically designed by a Scientific Committee appointed by the Italian Ministry of Health was used to appraise the 19 RPPs. Descriptions of the structure of the RPPs and the methodology used to develop the tool are presented elsewhere [15]. Briefly, RPPs have two core sections: i) a Strategic Framework Section, which deals with the analysis of the regional context, identification of local needs, description of regional health planning and definition of priorities for the RPP 2010–2012; and ii) an Operational Plan Section, in which projects are developed as a consequence of the planning choices set out in the Strategic Framework Section [15]. The appraisal tool reflects the structure of the RPPs and thus also has two sections: i) a descriptive analysis of the RPP, focused mainly on the Strategic Framework Section; and ii) an analysis of the projects included in the Operational Plan Section of the RPP [16]. The analysis of each RPP was carried out by working groups composed of at least two independent members, with discrepancies resolved by discussion.

Data analysis was carried out with two objectives: i) to describe to what extent RPP projects consider health inequalities and specific public health issues in vulnerable populations; ii) to identify regional characteristics that may be associated with the different levels of attention paid to health inequalities in RPP projects. Projects are required to match one or more of 22 general lines of intervention grouped into four macroareas (Predictive medicine, Universal prevention, Prevention in high risk groups and Prevention of complications and recurrence of chronic diseases).

The level of attention to inequalities in RPP projects was evaluated using the following three Yes/No questions taken from a specific tool for the assessment of the attention to equity of prevention projects based on international guidelines [17–19] and adapted to the Italian context [20]: i) Did the project include activities aimed at solving public health problems in vulnerable populations? (i.e., did the project discuss on the burden of disease in disadvantaged populations and include activities to reduce it?) [17]; ii) Was the project aimed at improving the access of vulnerable groups to health services? (i.e., did the project discuss barriers to implementation in disadvantaged populations, and identify strategies to overcome these barriers?) [17]; iii) Did the project include an evaluation of its impact on vulnerable populations? (i.e., did the project contain plans for monitoring disadvantaged groups according to place of residence, race, occupation, gender, religion, education, socioeconomic status, or social network and capital?) [17]. A fourth question (iv) was added to identify whether projects were specifically aimed at the reduction of health inequalities.

To identify regional characteristics associated with the degree of consideration of health inequalities, a dichotomous variable was obtained by assigning to each project a value of 1 if there was a positive answer to at least one of the four questions described above. Statistical analysis was carried out by univariate and multiple logistic regression analyses.

Student’s *t*-test (for continuous variables) and *χ*^2^ test (for categorical variables) were used to evaluate the association between attention to inequalities in RPP projects and the following set of variables, some of which are based on the most reliable institutional indicators for the description of the socioeconomic status of the Regions:

i) macroarea of intervention (Predictive medicine, Universal prevention, Prevention in high-risk groups and Prevention of complications and recurrence of chronic diseases);
ii) geographic area (North, Center, South and Islands);
iii) quality of the Strategic Framework section of the RPP. In order to adjust the model for the quality of the RPPs, we considered 21 items of the appraisal tool of the Strategic Framework Section of the RPP. A principal component analysis (PCA) was performed to reduce the number of items. The following 10 items were retained: regional demographical context clearly described; regional socio-economic context clearly described; regional epidemiological context clearly described; organizational context clearly described; organizational needs clearly stated; epidemiological needs clearly stated; information on regional health programming provided; criteria used to establish the priorities clearly stated; all identified needs and priorities translated into specific projects; the RPP reported the results of the previous RPP. The 0–10 summary score was calculated and included in the model as a dichotomous variable (high if above the median, low-medium if below). The complete methodology is described in detail elsewhere [21];
iv) regional Gini index in 2010 [22] (categorized in quartiles);
v) presence of a formal Recovery Plan in the Region, imposed by the Central Government as a consequence of a regional structural deficit in the health care budget to establish objectives and strategic actions by which Regions might restore financial equilibrium and remove determinants of structural imbalance [21];
vi) regional health care expenditure (RHCE) as a percentage of Regional Gross Domestic Product (RGDP) in 2010 (continuous) [22];
vii) percentage of migrants of the total population in the Region in 2010 (continuous) [22];
viii) regional deprivation index in 2010 (continuous) [22];
ix) regional score of civicness, using the most updated score derived by Putnam et al. [23], which, based on a series of indexes extracted by the National Health Interview Survey 2007 [24], is intended to measure the so-called ‘linking social capital’ of the Region [25]; this score describes the vertical relationships connecting individuals, or the social networks to which they belong, to people or groups who are in positions of political power or status [24]. This aspect of civicness plays a crucial role in the economic growth of [26], the increase of trust in [27] and the performance of the institutions concerned [28].

A multiple logistic regression model was built to identify the regional characteristics associated with attention to health inequalities in RPPs projects. All the aforementioned variables were included in the model. Interaction terms were tested using a cut-off significance level of 0.15 and robust standard errors were estimated to adjust for the regional (cluster) effect. Tolerance and variance inflation factor (VIF) were measured to estimate multicollinearity among independent variables. Adjusted odds ratios (ORs) and 95 % confidence intervals (CIs) were calculated. Statistical analysis was performed using STATA statistical software, version 12.0 (Stata Corp. LP, College Station, TX. USA, 2011).

## Results

Of the 702 projects included in the 19 RPPs developed by the Italian Regions, only 56 (8.0 %) addressed at least one of the four issues related to health inequalities (Table 1). Emilia Romagna Region addressed the highest percentage of projects in its RPP to the problems of health inequalities (21.8 %), while at the other extreme two Regions (Basilicata and Sardegna) developed no projects of this type. Across all Regions, only 13 projects (1.9 %) were specifically aimed at the reduction of inequalities, six of which were developed by Emilia Romagna. The majority of the projects included activities aimed at solving public health problems relevant to vulnerable populations (42, 6.0 %) and aimed at improving the access of vulnerable populations to health services (38, 5.4 %). Only six Regions (Liguria, Emilia Romagna, Toscana, Umbria, Marche and Calabria) developed projects (21 overall, 3.0 %) that evaluated their impact on vulnerable populations (Table 1).

**Table 1:** Descriptive analysis of projects included in the Regional Prevention Plans (RPPs) that addressed the reduction of inequalities and specific public health issues concerning vulnerable populations

Region	Total number of project included in the RPP	Projects specifically aimed at the reduction of inequalities	Projects that included activities aimed at solving public health problems in vulnerable populations	Projects aimed at improving the access of vulnerable groups to health services	Projects that had an impact evaluation on vulnerable population	Total number of project addressing at least one of the four issues related to inequalities
		N (%)	N (%)	N (%)	N (%)	N (%)
NORTH						
Piemonte	64	2 (3.1)	4 (6.3)	2 (3.1)	0 (0)	4 (6.3)
Lombardia	23	0 (0)	3 (13.0)	2 (8.7)	0 (0)	3 (13.0)
A.P. Trento^a^	32	1 (3.1)	0 (0)	1 (3.1)	0 (0)	2 (6.2)
Veneto	71	0 (0)	1 (1.4)	0 (0)	0 (0)	1 (1.4)
FVG^b^	19	0 (0)	3 (15.8)	1 (5.3)	0 (0)	3 (15.8)
Liguria	40	1 (2.5)	6 (15.0)	6 (15.0)	7 (12.5)	6 (15.0)
Emilia Romagna	55	6 (10.9)	9 (16.4)	9 (16.4)	9 (16.4)	12 (21.8)
CENTER						
Toscana	49	1 (2.0)	2 (4.1)	2 (4.1)	2 (4.1)	2 (4.1)
Umbria	27	0 (0)	2 (7.4)	1 (3.7)	1 (3.7)	2 (7.4)
Marche	30	0 (0)	2 (6.7)	5 (16.7)	0 (0)	5 (16.7)
Lazio	22	1 (4.6)	2 (9.1)	2 (9.1)	1 (4.6)	2 (9.0)
SOUTH						
Abruzzo	21	0 (0)	0 (0)	1 (4.8)	0 (0)	1 (4.8)
Molise	31	0 (0)	1 (3.2)	0 (0)	0 (0)	1 (3.2)
Campania	42	0 (0)	3 (7.1)	5 (11.9)	0 (0)	6 (14.3)
Puglia	39	0 (0)	1 (2.6)	0 (0)	0 (0)	1 (2.6)
Basilicata	19	0 (0)	0 (0)	0 (0)	0 (0)	0 (0)
Calabria	70	1 (1.4)	2 (2.9)	1 (1.4)	3 (4.3)	4 (5.7)
ISLANDS						
Sardegna	18	0 (0)	0 (0)	0 (0)	0 (0)	0 (0)
Sicilia	30	0 (0)	1 (3.3)	0 (0)	0 (0)	1 (3.3)
TOTAL	702	13 (1.9)	42 (6.0)	38 (5.4)	21 (3.0)	56 (8.0)

^a^ *A.P. Trento* Autonomous Province of Trento

^b^ *FVG* Friuli Venezia-Giulia

Univariate and multivariate analyses allowed us to examine the association of attention to inequalities in RPP projects with several variables. Projects addressing health inequalities were more frequent in the macroarea of ‘prevention in high-risk groups’, and were associated with a higher quality of the Strategic Plan section of the RPP. Moreover, the percentage of migrants in the regional population was higher for projects which devoted attention to inequalities. Geographic macroarea, regional Gini index, presence of a Recovery plan in the Region, regional healthcare expenditure as a percentage of RGDP and deprivation index showed no significant impact according to the univariate analysis (Table 2).

**Table 2:** Analysis of projects included in the Regional Prevention Plans (RPPs) that addressed the reduction of inequalities and specific public health issues concerning vulnerable populations, according to selected variables

Variables	Projects with attention to inequalities^a^		
	No	Yes	pV
	N. (%)	N. (%)	
Macroarea of intervention			
Predictive medicine	34 (97.1)	1 (2.9)	<0.001*
Universal prevention	415 (94.3)	25 (5.7)	
Prevention in populations at risk	163 (84.9)	29 (15.1)	
Tertiary prevention^b^	34 (97.1)	1 (2.9)	
Geographic area			
North	274 (90.1)	30 (9.9)	0.198
Center	117 (91.4)	11 (8.6)	
South	208 (93.7)	14 (6.3)	
Islands	47 (97.9)	1 (2.1)	
Quality score of the Strategic Plan section of the RPP^c^			
Low/medium	448 (94.3)	27 (5.7)	0.001*
High	198 (87.2)	29 (12.8)	
Regional Gini index			
1^st^ quartile	142 (93.4)	10 (6.6)	0.172
2^nd^ quartile	193 (91.9)	17 (8.1)	
3^rd^ quartile	145 (87.4)	21 (12.6)	
4^th^ quartile	162 (93.1)	12 (6.9)	
Recovery Plan in the Region			
No	348 (90.9)	35 (9.1)	0.213
Yes	298 (93.4)	21 (6.6)	
	Mean (SD)	Mean (SD)	
Regional health care expenditure as % of the RGDP^d^			
(continuous)	8.1 (2.0)	7.7 (1.9)	0.121
Percentage of migrants in the Region			
(continuous)	7.1 (3.4)	8.0 (3.2)	0.046**
Deprivation index			
(continuous)	15.6 (7.0)	14.3 (7.1)	0.203

^a^See text for definition

^b^Tertiary prevention: Prevention of complications and recurrence of diseases

^c^ *RPP* Regional Prevention Plan

^d^ *RGDP* Regional Gross Domestic Product

*pV < 0.05 (Chi-square test)

**pV < 0.05 (Student’s *t* test)

Multiple logistic regression analysis confirmed the statistically significant association of the level of attention to inequalities in projects with the macroarea of intervention ‘prevention in high-risk groups’, with higher quality of the Strategic Plan section of the RPP and with higher percentage of migrants in the Region (Table 3). Moreover, projects that considered health inequalities were significantly more likely to be developed by Northern Regions, and by Regions with a higher RHCE as a percentage of RGDP and with a lower level of civicness. The variable ‘regional deprivation index’, originally included in the model, was excluded due to collinearity (VIF: 30.22; tolerance: 0.03). No other variables showed critical tests’ results (Table 3).

**Table 3:** Results of the multiple regression model investigating possible predictors of the attention to inequalities of projects included in the Regional Prevention Plans (RPPs)

	OR	95 % CI	pV
Macroarea of intervention			
Universal prevention (reference)	1.00	–	–
Predictive medicine	0.40	0.04–3.74	0.420
Prevention in high risk groups	3.14	1.73–5.73	<0.001
Prevention of complications and recurrence of chronic diseases	0.45	0.05–4.08	0.476
Geographic area			
North (reference)	1.00	–	–
Center	0.52	0.29–0.94	0.031
South	0.69	0.22–2.21	0.534
Islands	0.12	0.03–0.51	0.004
Quality score of the Strategic Plan section of the RPP^a^			
Low (reference)	1.00	–	–
High	2.51	1.42–4.43	0.002
Regional Gini index			
1^st^ quartile (reference)	1.00	–	–
2^nd^ quartile	1.25	0.45–3.48	0.671
3^rd^ quartile	0.86	0.33–2.25	0.757
4^th^ quartile	1.81	0.56–5.92	0.324
Recovery plan in the Region			
No	1.00	–	–
Yes	1.22	0.38–3.88	0.735
Regional health care expenditure as % of RGDP^b^			
Continuous	1.51	1.02–2.24	0.041
Percentage of migrants in the Region			
Continuous	1.48	1.08–2.03	0.016
Score of civicness			
Continuous	0.73	0.46–0.97	0.031

^a^ *RPP* Regional Prevention Plan

^b^ *RGDP* Regional Gross Domestic Product

Note: the variable ‘regional deprivation index’ was excluded due to collinearity

## Discussion

Multidisciplinary actions and policies which aim to counteract the various mechanisms that trigger inequalities and to reduce the impact of social inequality on health are among the priority actions of the health policy framework recently developed by the WHO regional office for Europe [29]. This approach needs strong health ministries, modern public health infrastructures and high-performing and equity-oriented health systems [30]. The urgent requirement for public health leaders able to understand the importance of programming targeted at the structural determinants of health has also been strongly emphasised in countries outside Europe, such as Canada [31] and Australia [32]. In the latter, a review found that government initiatives on prevention were more likely to be focused on individual behaviours linked to chronic diseases than on socioeconomic and cultural factors that drive such behaviours and, ultimately, disease outcomes [32].

Measures to combat inequalities have been adopted at the national level in Italy, but almost exclusively within the Health Service, rarely with the support of other areas and never as part of a comprehensive strategy. Instead of preventing the effect of social inequalities, in particular through health policies aimed at protecting the well-being of vulnerable groups, most actions focus on repairing the consequences of inequity, often without a clear and direct interest in reducing the gap in health between social groups [5].

The level of attention to the reduction of health inequalities showed by projects developed by Italian Regions within their RPPs was very low, since only 8.0 % of the projects showed specific attention to differences in health profiles among population subgroups and made proposals for the reduction of inequalities. Of particular interest is that these projects were more likely to be developed by Northern Regions, those with a higher percentage of migrants, those with a higher RHCE as a percentage of RGDP and those with a lower level of civicness. Moreover, projects that devoted attention to inequalities were more frequently in the macroarea of prevention in population at risk.

We found an association between a high percentage of migrants in the Region and a higher level of attention to inequalities in RPP projects. In Italy, resident foreigners are entitled to benefit from medical assistance under the same conditions as Italian citizens [33], while non-resident migrants are allowed to receive only urgent and/or essential medical care, including any preventive intervention aimed at protecting individual and public health [34]. In this context, the design of projects with preventive interventions specifically for migrant populations is crucial. Consistently, we found that the level of attention to health inequalities is higher on the prevention policy agenda in those Regions where migrants are numerous. It should be noted that, on arrival, migrants show, on average, better levels of health than native-born citizens, mainly because they are young and are seeking work [35–37]. This ‘healthy immigrant effect’, however, diminishes progressively as the immigrants assimilate the dominant culture and habits of the host nation, with their health status converging to that of native-born residents [38–40]. Therefore, improving the surveillance of the health of migrants, in particular through tailored programs of primary and secondary prevention among ethnic groups at higher risk, is essential from a public health perspective.

Central and Insular Regions and, to a lesser extent, Southern Regions developed projects with a lower level of attention to health inequalities. Geographical inequality in Italy is still one of the most discussed topics in Italian politics and public debates, and the gap between more developed Northern and less developed Southern Regions has not yet been closed. The most recent National Health Interview Survey, performed in Italy in 2013 [41], found an unequal distribution of health status, with better conditions in the Northern Regions than in Central and Southern Regions, the latter of which have the highest rates of hospitalization for both native Italians and immigrants, in particular for chronic diseases [42]. This North–south divide intensified after the decentralization of the Italian National Health System, such that a typical healthcare sector in the Southern Regions is less efficient and has a lower standard of care than counterparts in the Northern and Central Regions [43–45], mainly because of lower financial resources, but also due to cultural differences, socioeconomic development and technological infrastructure [23]. Therefore, in Central and Southern Regions, the implementation of preventive interventions aimed at improving living conditions and access to quality healthcare are strongly needed, since it has been proved that such a regional policy may reduce health disparities [46].

Our results show that Regions with higher RHCE as a proportion of GDP devote greater attention to health inequalities. We demonstrated previously that even lifestyle surveillance systems are more likely to be used in those Regions in this category [16]. However, tackling health inequalities could generate cost-saving health benefits in the long-term. The economic losses that health inequalities generate in Europe have been estimated recently and are substantial, both in absolute (€1000 billion) and in relative terms (9.4 % of GDP) [47]. Therefore, the design of strategies and interventions aimed at reducing health inequalities deserves to be placed higher on the European and Italian policy agenda [48].

Our finding that the less civic Regions give more attention to health inequalities in the prevention planning process would seem counterintuitive. As stated, we used a characteristic of social capital called ‘linking social capital’ to represent the capacity of the population to establish ‘vertical’ connection across power gradients, especially with representatives of institutions [49]. A possible explanation of this finding could be that Regions with less social capital have a stronger commitment to the welfare state. It has been argued that a strong welfare state has a negative impact on social capital, since it could replace social relationships, social trust, and civic activities [50, 51]. The basic argument is that both the need and incentives for the creation and maintenance of social contacts and civic activities decrease when the welfare state takes on responsibilities and duties that previously derived from people’s social networks and associations [52, 53].

Finally, the larger number of projects addressing health inequalities in the macroarea of ‘prevention in high risk groups’ deserves a comment. There is increasing evidence that screening programs intended to identify individuals with the highest levels of risk factors can widen health inequalities [9]. These interventions (e.g., behavioral change programs) require strong individual resources, both material and psychological, and thus tend to increase social inequalities [54–56]. This is particularly striking in cardiovascular disease prevention [9], but is also apparent in cancer prevention [57, 58]. Therefore, it seems consistent that projects within the macroarea of ‘prevention in high-risk groups’ gave more attention to health inequalities. By contrast, universal prevention projects may reduce social inequalities, in particular by means of structural strategies that work through changes in the wider social environment [9, 50].

## Conclusions

In conclusion, there is a low level of attention to health inequalities in the regional planning of prevention activities in Italy. This low level is particularly accentuated in Central and Southern Regions and is associated with a lower percentage of migrants, with a lower HCE as a percentage of RGDP and with a higher level of civicness. Projects addressing health inequalities are highly concentrated in the macroarea of ‘prevention in high risk groups’. The positive aspects of these results have been communicated to the Italian Ministry of Health and the new NPP 2014–2018 has taken account of the issues raised by this study, including the contrast in health inequalities among the five macro-objectives of the plan. In this respect, Italy might represent a good example of how public health research can support effective policies and interventions able to reduce health inequalities.

## Abbreviations

NPP: National Prevention Plan
RGDP: Regional Gross Domestic Product
RHCE: Regional Health Care Expenditure
RPP: Regional Prevention Plan
